# European Consortium for Lipodystrophies consensus definition and classification framework for monogenic lipodystrophy

**DOI:** 10.1111/joim.70157

**Published:** 2026-09-01

**Authors:** Robert K. Semple, Martin Wabitsch, Baris Akinci, Saif Alyaarubi, Marjoleine F. Broekema, Rebecca J. Brown, Giovanni Ceccarini, Carolina Cecchetti, Solaf M. Elsayed, Antía Fernández‐Pombo, Alessandra Gambineri, Ingrid M. Jazet, Lotte Kleinendorst, Konstanze Miehle, Elif A. Oral, Flavia Prodam, Ferruccio Santini, David B. Savage, Nadia Shaukat Ali, Ekaterina Sorkina, Magdalena Szopa, Marie‐Christine Vantyghem, Camille Vatier, Corinne Vigouroux, Julia von Schnurbein, David Araújo‐Vilar

**Affiliations:** ^1^ Institute for Neuroscience and Cardiovascular Research and Institute of Genetics and Cancer The University of Edinburgh Edinburgh UK; ^2^ Division of Pediatric Endocrinology and Diabetes Department of Pediatrics and Adolescent Medicine ENDO‐ERN Center for Rare Endocrine Diseases Ulm University Medical Center Ulm Germany; ^3^ Department of Technological Research Izmir Biomedicine and Genome Center Izmir Turkey; ^4^ Pediatric Endocrinology Oman Medical Specialty Board Muscat Oman; ^5^ Department of Human Genetics Amsterdam University Medical Center, University of Amsterdam Amsterdam the Netherlands; ^6^ National Institute of Diabetes and Digestive and Kidney Diseases National Institutes of Health Bethesda Maryland USA; ^7^ Obesity and Lipodystrophy Center Endocrinology Unit ENDO‐ERN Center for Rare Endocrine Diseases University Hospital of Pisa Pisa Italy; ^8^ Division of Endocrinology and Diabetes Prevention and Care Department of Medical and Surgical Sciences ENDO‐ERN Center for Rare Endocrine Diseases IRCCS Azienda Ospedaliero‐Universitaria di Bologna Bologna Italy; ^9^ Unit of Endocrinology and Metabolic Diseases G.B. Morgagni – L.Pierantoni Hospital Forlì Italy; ^10^ Medical Genetics Department Faculty of Medicine Ain Shams University Cairo Egypt; ^11^ UETeM‐Molecular Pathology Group Department of Psychiatry, Radiology, Public Health, Nursing and Medicine Instituto de Investigación Sanitaria Santiago de Compostela–Center for Research in Molecular Medicine and Chronic Diseases (IDIS–CIMUS) University of Santiago de Compostela Santiago de Compostela Spain; ^12^ Division of Endocrinology Department of Medicine Leiden University Medical Center Leiden the Netherlands; ^13^ Medical Department III—Endocrinology, Nephrology, Rheumatology University of Leipzig Medical Center Leipzig Germany; ^14^ Caswell Diabetes Institute and Metabolism Endocrinology and Diabetes Division Department of Internal Medicine University of Michigan Medical School Ann Arbor Michigan USA; ^15^ Unit of Endocrinology Department of Health Sciences University of Eastern Piedmont Novara Italy; ^16^ University of Cambridge Metabolic Research Laboratories Addenbrooke’s Hospital Cambridge UK; ^17^ Al Jalila Children's Speciality Hospital, Dubai Health Dubai United Arab Emirates; ^18^ Clinical Research Facility The Newcastle upon Tyne Hospitals NHS Foundation Trust Newcastle upon Tyne UK; ^19^ Department of Metabolic Diseases Jagiellonian University Medical College Krakow Poland; ^20^ Department of Endocrinology, Diabetology, Oncology and Metabolism Competence Center of Rare Diseases of Insulin‐Secretion and Insulin‐Sensitivity Lille University Hospital, PRISIS; INSERM U1190 Lille France; ^21^ APHP, Saint‐Antoine University Hospital National Reference Center for Rare Diseases of Insulin Secretion and Insulin Sensitivity Department of Endocrinology, Diabetology and Reproductive Endocrinology Endo‐ERN Assistance Publique Consortium and Sorbonne Université, Saint‐Antoine Research Center, Inserm UMR_S938 Paris France

**Keywords:** adipose tissue, classification, definition, genetic, lipodystrophy, monogenic

## Abstract

Lipodystrophy comprises a heterogeneous group of disorders characterized by reduced adipose tissue often associated with severe metabolic complications. Lipodystrophy may be genetic, acquired, or secondary to medical therapies initiated for other conditions. Despite major advances in understanding adipose tissue biology and in human genetic technologies, diagnosis of lipodystrophy is still commonly delayed or missed. Conversely, increasing use of next‐generation sequencing has led to proliferation of proposed new genetic causes of lipodystrophy with variable supporting evidence. To address these challenges, an international expert working group from the European Consortium for Lipodystrophies first developed a consensus definition of lipodystrophy as deficient development and/or inadequate maintenance of adipose tissue not attributable to nutritional deprivation or a systemic catabolic state. Anatomical adipose deficiency alone is generally insufficient for diagnosis, with evidence of adipose tissue dysfunction or pathology also usually required. Building on this definition, a framework for evaluating candidate monogenic lipodystrophy syndromes is suggested, based on the strength of human phenotypic, genetic, and biological evidence. Primacy was assigned to direct evidence from affected individuals, with genetic and biological evidence considered supportive but not determinative. This approach accommodates both the variable penetrance and expressivity characteristic of many lipodystrophy syndromes and potential discrepancies between human observations and experimental models. Although the proposed definition is applicable across all forms of lipodystrophy, the classification system described here is restricted to monogenic disorders. Together, these tools aim to improve clinical recognition, promote diagnostic consistency, and provide a robust and adaptable framework for evaluating emerging gene–disease associations in lipodystrophy.

## Graphical abstract

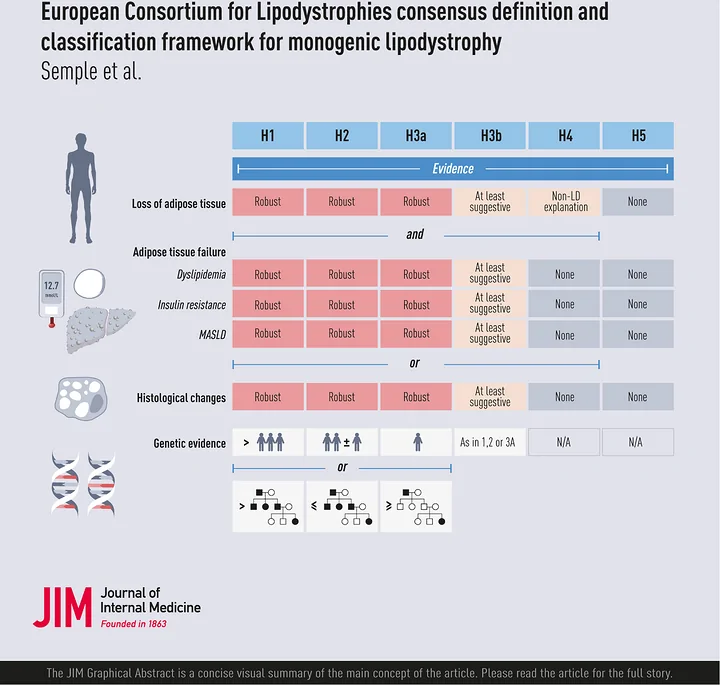

**Abbreviations**
AD: autosomal dominant
*AGPAT2*
: 1‐acylglycerol‐3‐phosphate O‐acyltransferase 2
*AIRE*
: autoimmune regulator
ANA: antinuclear antibodies
AR: autosomal recessive
AT: adipose tissue
B: supportive biological evidence
*BANF1*
: barrier to autointegration nuclear assembly factor 1
*BSCL2*
: lipid droplet biogenesis associated, seipin
C3/4: complement component 3/4
*CAV1*
: caveolin 1
*CIDEC*
: cell death inducing DFFA like effector c
ClinGen: clinical genome
dsDNA: double‐stranded deoxyribonucleic acid
DXA: dual energy x‐ray absorptiometry
ECLip: European Consortium for Lipodystrophies
FPLD1: familial partial lipodystrophy Type 1
GnoMAD: genome aggregation database
H: human evidence (clinical and genetic evidence)
HAART: highly active antiretroviral therapy
HDL: high density lipoprotein
HIV: human immunodeficiency virus
IR: insulin resistance
*LMNA*
: lamin A/C
*LPIN1*
: lipin 1
MASLD: metabolic dysfunction‐associated steatotic liver disease
MRI: magnetic resonance imaging
N/A: not applicable/not available
NGS: next‐generation sequencing
NIH: National Institutes of Health
*PCYT1A*
: choline‐phosphate cytidylyltransferase 1A
PD‐1: programmed cell death protein
*PIK3R1*
: phosphoinositide‐3‐kinase regulatory subunit 1
*POLD1*
: DNA polymerase delta 1, catalytic subunit
*POLR3 A* or *B*
: RNA polymerase III subunit A or B
*PSMB10*
: proteasome 20S subunit beta 10
SHORT syndrome: syndrome associated with short stature, hyperextensibility, ocular depression, rieger anomaly, teething delay
TNFα: tumor necrosis factor alpha

## An introduction to lipodystrophy—“obesity” complications without obesity

Obesity—excess adipose tissue—is a major global public health threat, closely associated with premature mortality and multisystem morbidities including Type 2 diabetes, metabolic dysfunction‐associated steatotic liver disease (MASLD), accelerated atherosclerosis, subfertility, and cancer [1]. Much less heralded is the fact that a near‐identical array of associations, often severe, is also seen in people with reduced ability to develop and/or maintain adipose tissue—a condition known as lipodystrophy [2].

The disease associations of obesity, taken at face value, appear to indicate that high adiposity is harmful. However, the fact that these same associations are also seen in people living with pathologically reduced adipose tissue argues for a more nuanced relationship between adiposity and disease. Lipodystrophy is thus not only an important disorder to recognize and treat in its own right; it also offers key insights into the mechanisms mediating the links among obesity and major common diseases [3].

## Historical perspectives on lipodystrophy

The first descriptions of lipodystrophy date back to the 19th century and largely concerned forms now recognized as acquired or autoimmune disorders. The syndrome later known as Barraquer–Simons disease was characterized by progressive loss of subcutaneous adipose tissue beginning in the face and upper body [4, 5]. A major conceptual advance occurred in 1946, when Lawrence described a woman with severe adipose deficiency who nevertheless exhibited insulin‐resistant diabetes, hypertriglyceridemia, and hepatic steatosis [6]. Lawrence proposed that the inability of adipose tissue to appropriately store energy was central to the pathogenesis of these metabolic complications [7]. Although formulated decades before modern concepts of adipose biology emerged, this insight anticipated current understanding of adipose tissue failure as a driver of systemic metabolic disease.

During the second half of the 20th century, additional forms of lipodystrophy were recognized, including congenital generalized lipodystrophy [8, 9] and familial partial lipodystrophy [10, 11]. These observations established lipodystrophy as a clinically heterogeneous group of disorders sharing a common biological theme: deficient development, maintenance, or function of adipose tissue.

## Genetic and biological basis of lipodystrophy

The modern era of lipodystrophy research began at the turn of the 21st century through the convergence of human genetics and experimental biology. Landmark studies in mice demonstrated that near‐complete ablation of white adipose tissue resulted in severe insulin resistance (IR), hepatic steatosis, and premature mortality, closely mirroring the phenotype observed in human generalized lipodystrophy [12, 13]. These observations reinforced the concept that adipose tissue is not merely a passive energy store but a critical endocrine and metabolic organ.

Shortly thereafter, genetic studies identified pathogenic variants in *LMNA* as a cause of familial partial lipodystrophy [14, 15] and biallelic variants in *BSCL2* [16] and *AGPAT2* [17] as causes of congenital generalized lipodystrophy (see list of abbreviations). Subsequent discoveries implicated numerous additional genes involved in adipocyte differentiation, lipid droplet biology, membrane phospholipid synthesis, nuclear envelope integrity, and cellular stress responses [18]. Collectively, these studies transformed lipodystrophy from a largely descriptive clinical entity into a biologically coherent group of disorders rooted in adipose tissue dysfunction.

Lipodystrophy is now recognized as encompassing genetic, acquired, and secondary forms. Acquired lipodystrophies include autoimmune syndromes such as acquired generalized and acquired partial lipodystrophy, whereas secondary forms may occur in association with specific treatments such as antiretroviral therapy or after stem cell transplantation. Although these conditions differ in etiology, they share the defining feature of pathological adipose tissue deficiency and frequently result in similar metabolic complications. The present work focuses specifically on monogenic lipodystrophies while recognizing the broader spectrum of lipodystrophic disorders.

## Current challenges in diagnosis and classification

Despite major advances in understanding lipodystrophy, substantial challenges remain. The first is underrecognition in clinical practice. Patients may initially present to endocrinologists, diabetologists, hepatologists, cardiologists, geneticists, pediatricians, or other specialists, many of whom have limited exposure to lipodystrophy during training. Consequently, diagnosis is frequently delayed for years or even decades, resulting in preventable metabolic and organ‐specific complications.

To raise clinical awareness, improve clinical care, and support lipodystrophy research, the European Consortium for Lipodystrophies (ECLip; https://www.eclip‐web.org/) was founded in 2014. ECLip now spans 30 countries, hosts a rapidly growing clinical registry (https://eclip‐registry.com) [19, 20], and counts 175 clinicians and clinician‐scientists among its membership. Despite concerted efforts by ECLip and other professional networks, in partnership with patient advocacy groups (https://aelip.es/, https://lipodystrophyuk.org, https://aflip.org, https://ailip.it, https://netlip.org, https://lipodystrophyunited.org) progress in disseminating knowledge of lipodystrophy among the specialists who encounter its manifestations has gained only moderate traction. This is a major problem for affected people, as delayed diagnosis leads directly to avoidable harm that accrues before optimal management is instituted, as evidenced in recent cross‐sectional surveys [21, 22, 23].

A second challenge arises from the rapid expansion of genomic medicine. Widespread access to next‐generation sequencing has accelerated the discovery of candidate disease genes but has also led to increasingly variable use of the term “lipodystrophy” in the literature [24, 25, 26, 27, 28]. In some reports, reduced adipose tissue mass is interpreted as evidence of lipodystrophy despite limited or no evidence for primary adipose tissue dysfunction, creating uncertainty regarding the validity of proposed disease associations. Clinical diagnosis in lipodystrophy can thus be viewed as suffering both from lack of sensitivity—that is, failure to recognize bona fide lipodystrophy clinically—whereas lipodystrophy research sometimes shows lack of specificity—that is, imprecise use of the “lipodystrophy” label.

A related challenge concerns how different forms of evidence should be integrated when evaluating candidate disease genes. Existing gene–disease validity frameworks frequently combine clinical, genetic, and experimental evidence into a single overall assessment. However, experience from lipodystrophy suggests that such evidence may not always be concordant. Some genes produce striking adipose phenotypes in experimental models without causing lipodystrophy in humans [29, 30], whereas several well‐established human lipodystrophy syndromes are only partially recapitulated in model systems [31, 32, 33]. These observations argue for maintaining distinct evidential tiers rather than collapsing them into a single score.

To address these challenges, the European Consortium for Lipodystrophy convened an international expert working group. Its objectives were (i) to develop a clinically applicable definition of lipodystrophy grounded in adipose tissue biology; (ii) to establish a framework for evaluating the strength of evidence supporting candidate monogenic lipodystrophy syndromes; and (iii) thus to facilitate both earlier clinical recognition and more rigorous interpretation of emerging genetic discoveries. A particular focus was on subtypes with systemic metabolic complications, as it is in these groups that the greatest harm arises from delayed diagnosis.

## Development of the ECLip consensus framework

### Composition of working group

The working group was composed of clinically active physicians with a special interest in lipodystrophy who volunteered to contribute from the wider ECLip network. Members were drawn mostly from clinical endocrinology/diabetes (84%) and clinical genetics (12%), spanning adult (68%) and pediatric (28%) medicine. Overall, 80% of the group also currently conducts translational or fundamental lipodystrophy research. The group spanned 14 countries, largely in Europe, but with representation also from the USA, Middle East, and North Africa. Several of the group were leaders of national referral centers for lipodystrophy and related disorders.

### Scope of study

The study aimed to yield a generalizable definition of lipodystrophy and a framework for evaluating candidate monogenic lipodystrophy. Acquired forms of lipodystrophy were set aside for future consideration using a parallel approach, whereas oligogenic familial partial lipodystrophy Type 1 (FPLD1) [34, 35], which may affect 10% or more of the general adult population, will also be the subject of future work.

### Development of consensus definition

The consensus definition was developed through a series of virtual meetings, coupled with discussion and iterative refinement between meetings, all conducted between March 2024 and December 2025, using a process presented to the 2025 annual ECLip meeting (https://site‐752516.mozfiles.com/files/752516/ECLIP2025‐1.pdf) [36]. A final definition was reached that was unanimously approved by the working group.

### Identification of candidate lipodystrophy subtypes

The selection of candidate monogenic lipodystrophy subtypes was based on the extensive experience and expertise of the working group members, supported by a literature review. A systematic review and classification of all proposed forms of lipodystrophy will be the subject of subsequent work. In the current article, we describe selected disease entities only to exemplify the underpinning principles and practical use of this consensus framework.

### Process developed to evaluate strength of evidence for candidate monogenic lipodystrophy

Subsequent to development of the definition of lipodystrophy, the working group codified pragmatic rules to classify all candidate monogenic lipodystrophies into ranked categories depending on strength of evidence. Provisional criteria were developed in a series of group discussions before testing as a pilot exercise in a subset of candidate lipodystrophies. Lessons from this pilot exercise were discussed before refinement of the rules, which were then unanimously agreed upon.

## Proposed definition of lipodystrophy

Deliberation began with agreement that lipodystrophy denotes anatomic and functional impairment of adipose tissue. It was further recognized that such impairment can reflect failure of development of adipose tissue, failure to maintain adipose tissue, or both. Failure of adipose development usually means a defect in the process of adipocyte differentiation, whereas failure to maintain adipose tissue can reflect pathological destruction of adipose tissue, for example, through autoimmune attack, or impaired resilience in the face of naturally occurring endogenous insults such as DNA damage. It was agreed that lipodystrophy means “deficient development and/or inadequate maintenance of adipose tissue.”

Whole body adiposity is straightforward to quantify using techniques such as dual‐energy x‐ray absorptiometry, with values referenced to control populations matched for sex, age, and pubertal status. However, while generalized lipodystrophy may result in extremely low whole‐body adiposity, for most forms of lipodystrophy, adiposity lies in the general population range, albeit usually toward the lower end. Global measures of body fat content thus cannot be taken to indicate lipodystrophy when considered alone.

Adipose tissue is highly plastic and responsive to energy balance. It expands when there is a need to increase energy storage capacity to accommodate energy intake that exceeds energy expenditure. When energy intake is low relative to energy expenditure, adipose tissue may be lipid‐depleted and not easily detectable by physical examination or imaging, yet still capable of expansion should energy balance change. Such physiological adipose shrinkage is by far the commonest reason for reduced adipose tissue mass, seen in chronic undernutrition, endurance athletes, and pathological catabolic states such as hyperthyroidism, severe burns, uncontrolled diabetes, anorexia nervosa [37], or cancer cachexia. These considerations led to inclusion of the qualification that “underdevelopment or loss of adipose tissue is not due to nutritional deprivation or a systemic catabolic state.”

A crucial discriminator of pathological lack of adipose tissue lies in consideration of adipose function as well as anatomy. In physiological depletion of adipose tissue, the metabolic profile should be concordant, featuring low insulin concentrations, lack of MASLD, and low serum triglyceride concentrations, with no evidence of clinical features of IR such as acanthosis nigricans. When a person with visibly low adiposity instead has clinical or biochemical evidence of IR, usually with a low serum high density lipoprotein (HDL)‐cholesterol, high serum triglyceride concentration, and MASLD, this mismatch should immediately lead to suspicion of lipodystrophy. This may be a particularly valuable clue in men, in whom obvious muscularity and prominent veins raise fewer concerns among patients or clinicians.

The plasma concentration of adiponectin can also be very helpful in discriminating physiological leanness and lipodystrophy. Adiponectin is a stable, highly abundant adipocyte‐derived plasma protein that paradoxically increases on physiological decreases in adipose tissue mass [38], even in advanced stages of undernutrition [39], whereas it is low in lipodystrophy [40]. A high adiponectin concentration in a person with low adipose mass is thus a strong argument against lipodystrophy [37].

An important caveat to the “rule” that mismatch between low adiposity and metabolic features of obesity is the hallmark of lipodystrophy is that the converse is not true—that is, lack of signs of metabolic distress—or “adipose failure”—in someone with anatomical deficiency of adipose tissue does not exclude lipodystrophy. This is because severe negative energy balance depletes adipose tissue triglyceride stores and removes demand for further storage. This masks the effects of functional deficiency. Put most succinctly, a severely undernourished person with lipodystrophy is likely to look closely similar to a severely undernourished person without lipodystrophy. These considerations were captured in the definition by the statement “without evidence of adipose functional insufficiency, and/or histological evidence of adipose pathology in affected depots, it is difficult to diagnose lipodystrophy with confidence in lean people.”

It was further recognized that white adipose tissue, though histologically relatively bland, is highly heterogeneous and regionalized, with corresponding differences in anatomical patterns of lipodystrophy, some reflecting differences in embryological origin of different adipose tissue depots [41, 42, 43, 44, 45]. Loss of adipose tissue in some regions often leads to compensatory expansion elsewhere; indeed, people with lipodystrophy may be diagnosed with obesity by conventional criteria [43]. This was captured in the statement “regional adipose tissue loss may be associated with expansion of adipose depots in other areas of the body.”

The differences in patterns of loss across different anatomical adipose depots in partial lipodystrophies yield important anatomical clues to lipodystrophy. Although measures of global adipose content are poor indicators of lipodystrophy when considered alone, measures that capture differences among adipose depots have more discriminating value. Recent guidelines and expert consensus statements thus recommend the use of measures such as lower limb fat percentage, the fat mass ratio (trunk‐to‐limb fat ratio), or selected skinfold measurements as supportive tools for the clinical diagnosis of lipodystrophy [34, 46, 47, 48, 49]. Anatomical heterogeneity across the many forms of FPLD, and variation in fat content and distribution based on sex, age, and methodology, do not yet allow generalized use of anatomical metrics, however [46, 50, 51].

The proposed definition notes that lipodystrophy that is highly localized anatomically, whether idiopathic or related to local damage, is generally apparent to the eye, but most commonly is not of sufficient extent to cause systemic features of “adipose failure.” Such subtypes of lipodystrophy, whereas they may be aesthetically distressing, are not associated with the large burden of cardiometabolic and other systemic complications that arise when diagnosis of more widespread lipodystrophy is delayed.

1 **Box 1. Consensus definition of lipodystrophy**

**1. Core definition**

Lipodystrophy is defined as deficient development and/or inadequate maintenance of adipose tissue that is not attributable to nutritional deprivation or a systemic catabolic state.

**2. Anatomical manifestations**

The disorder may be generalized or regional in distribution.

Regional adipose tissue loss may coexist with expansion of adipose depots in other anatomical regions.

Global adipose tissue mass alone is insufficient to establish the diagnosis, particularly in partial forms of lipodystrophy.

To our current knowledge this loss of adipose tissue is irreversible.

**3. Adipose tissue dysfunction (**“**adipose failure”)**

Evidence of adipose tissue dysfunction is a hallmark of most clinically significant forms of lipodystrophy.

This most commonly manifests as one or more of insulin resistance and/or diabetes mellitus, hypertriglyceridemia, low HDL‐cholesterol, metabolic dysfunction‐associated steatotic liver disease (MASLD).

Histological evidence of adipose tissue pathology may also support the diagnosis.

**4. Distinguishing lipodystrophy from physiological leanness**

Reduced adipose tissue mass due to undernutrition, intense physical training, or systemic catabolic illness should not be considered lipodystrophy.

In lean individuals, diagnosis of lipodystrophy generally requires evidence of adipose tissue dysfunction and/or histological evidence of adipose pathology.

**5. Localized lipodystrophy**

Localized forms of lipodystrophy are recognized exceptions.

In these disorders, adipose tissue loss is usually anatomically obvious, but as it is confined to limited anatomical regions, it may not be extensive enough to produce systemic metabolic abnormalities.

**6. Syndromic lipodystrophy**

Lipodystrophy may occur as an isolated disorder or as part of a broader syndromic condition.

Associated manifestations vary according to the underlying genetic or acquired etiology.

Finally, lipodystrophy can be part of a more complex syndrome, often co‐occurring with one or more other developmental abnormalities. Metabolic and lipodystrophic manifestations of complex syndromes, though they may confer substantial long‐term complications, are often less well documented than other features. The final agreed definition is shown in Box 1. A diagnostic algorithm of lipodystrophy syndromes is shown in Fig. 1.

**Fig. 1:**
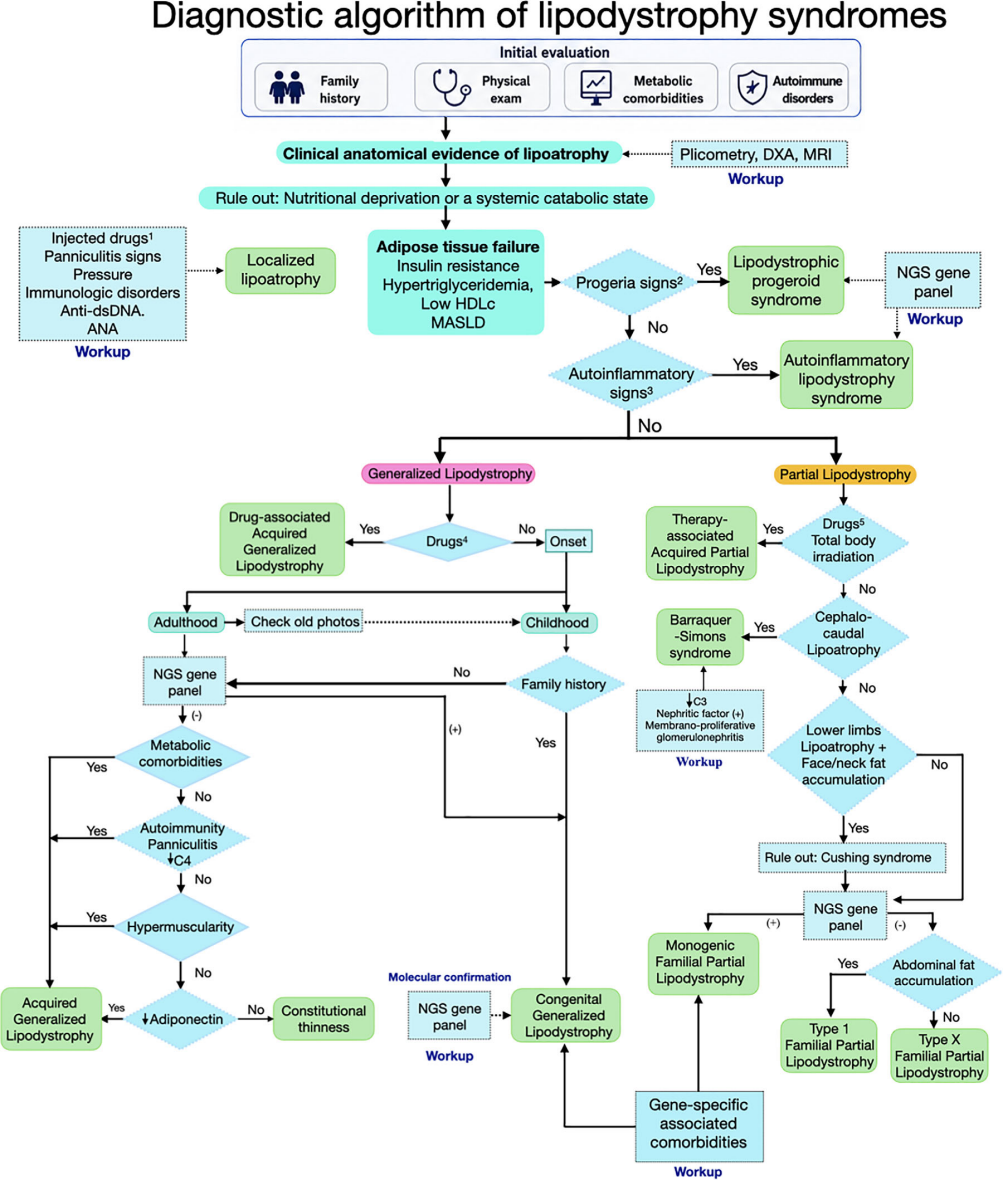
Diagnostic algorithm of lipodystrophy syndromes. The figure depicts how a specific lipodystrophy syndrome can be diagnosed in a patient fulfilling the criteria for lipodystrophy. (1) Injected drugs: insulin; injectable corticosteroids, particularly intralesional triamcinolone, as well as intramuscular corticosteroids; pegvisomant, long‑acting exenatide, anti‑TNF‑α biologics, recombinant growth hormone/somatrogon, subcutaneous medroxyprogesterone; and, with older or less robust reports, amikacin, benzathine penicillin, methotrexate, bleomycin, iron, heparin, vaccines, and growth factors. (2) Progeria signs: Growth delay, short stature, sarcopenia, alopecia or very sparse hair, thin, atrophic or prematurely aged skin sometimes with abnormal pigmentation, micrognathia/mandibular hypoplasia and a beaked nose, “bird‑like” facies, joint contractures and stiffness, acral osteolysis, bone deformities, osteopenia or osteoporosis, nail dystrophy, and high‑pitched voice, among the most prominent features. (3) Autoinflammatory signs: Recurrent fever; cutaneous lesions such as erythematous‑edematous or violaceous plaques, pernio‑like rash and nodular panniculitis; joint contractures, arthralgias, morning stiffness, and muscle atrophy; microcytic or hypochromic anemia and elevated acute‑phase reactants, with polyclonal hypergammaglobulinemia. (4) Drugs: Anti‑PD‑1 immune checkpoint inhibitors, particularly nivolumab and pembrolizumab; cases have also been reported with combined ipilimumab–nivolumab therapy. HIV antiretroviral drugs. (5) Drugs: HIV antiretroviral drugs, particularly protease inhibitors and HAART as a whole. ANA, antinuclear antibodies; C3/4, complement component 3/4; dsDNA, double‐stranded deoxyribonucleic acid; DXA, dual energy x‐ray absorptiometry; HAART, highly active antiretroviral therapy; HDL, high density lipoprotein; HIV, human immunodeficiency virus; MASLD, metabolic dysfunction‐associated steatotic liver disease; MRI, magnetic resonance imaging; NGS, next‐generation sequencing; PD‐1, programmed cell death protein.

## Evidence framework for candidate monogenic forms of lipodystrophy

Having established a consensus definition of lipodystrophy, the next objective was to develop a framework for evaluating the growing number of reported gene–disease associations. Initial pilot classification of candidate disorders highlighted three recurrent challenges: distinguishing primary lipodystrophy from adipose depletion secondary to negative energy balance, assessing genotype–phenotype relationships in disorders with variable penetrance and expressivity, and interpreting biological evidence from experimental models that may not faithfully reproduce human disease.

The resulting framework adopts a hierarchical approach in which evidence derived from affected individuals forms the foundation of classification. Human evidence is assessed according to the presence of anatomical adipose tissue deficiency together with evidence of adipose tissue dysfunction or pathology, consistent with the proposed definition of lipodystrophy. Adipose tissue dysfunction most commonly manifests as IR, dyslipidemia, and MASLD, although histological evidence of adipose pathology may also satisfy this criterion.

Genetic evidence is subsequently integrated with phenotypic observations. Evaluation takes account of the particular challenges inherent to lipodystrophy, including reduced fertility in many affected individuals, limited family sizes, and the influence of age, sex, and energy balance on disease expression. These factors may complicate interpretation of genotype–phenotype co‐segregation and reduce the amount of informative human genetic evidence available for some disorders.

Finally, supporting biological evidence is incorporated in two tiers. The higher tier (**B1**) requires experimental findings directly supporting a role for the implicated gene in adipose tissue development, maintenance, or function, whereas the lower tier (**B2**) includes evidence that gene function is altered without direct demonstration of adipose‐specific consequences. The absence of supporting biological evidence is designated **B0** (Table 1).

**Table 1:** Suggested classification scheme for candidate monogenic lipodystrophies.

	H1—confirmed	H2—probable	H3A—possible	H3B—possible	H4—no evidence	H5—refuted
**Phenotype** (essential)	Robust anatomic evidence of AT deficiency^a^ **AND** Robust clinical/metabolic evidence of “adipose failure”^b^ and/or histological evidence of adipose pathology			At least suggestive anatomic evidence of AT deficiency^c^ **AND** At least suggestive clinical/metabolic evidence of “adipose failure”^c^ and/or histological evidence of adipose pathology	Reduced AT with plausible non‐lipodystrophic explanations **AND** No clinical/metabolic evidence of “adipose failure” and/or histological evidence of adipose pathology	Ample AT with no regional deficiency **AND** No clinical/metabolic evidence of “adipose failure” and/or histological evidence of adipose pathology
**Genetic evidence** ^d^ (essential for candidate genetic subtypes only)	>3 unrelated kindreds OR one kindred with >four affected individuals and clear co‐segregation of genotype/phenotype^e^	Two to three affected individuals, with clear co‐segregation of genotype/phenotype (in one kindred up to four affected individuals)^e^	Single affected individual, or >one affected with imperfect co‐segregation of genotype and phenotype^e^	As in 1, 2 or 3A	N/A	N/A
Supportive **biological evidence** ^f^	**B1**. Animal model(s) recapitulating human phenotype and/or cellular model(s) showing impaired adipogenesis/lipogenesis/lipolysis **B2**. Evidence that function of gene is impaired in cell or biochemical studies, but without lipodystrophy‐specific abnormalities proven **B0**. No experimental evidence					

**Abbreviations:** AT, adipose tissue; B, supportive biological evidence; GnoMAD, genome aggregation database; H, human evidence (clinical and genetic evidence); HDL, high density lipoproteins; MASLD, metabolic dysfunction‐associated steatotic liver disease; N/A, not applicable.

^a^ For example, photographs, skinfold measurements, or imaging showing global or regional adipose deficiency.

^b^ That is, insulin resistance WITH low HDL‐cholesterol, high triglycerides, or MASLD and/or clear histological abnormalities in residual AT.

^c^ Human anatomical or functional evidence for lipodystrophy, but not both, may be robust in this category.

^d^ In evaluating potentially causal genetic variants, it is essential that allele frequency and evidence of evolutionary constraint, for example, from GnoMAD (105), are taken into account and weighed against prevalence of the syndrome concerned.

^e^ Perfect segregation is taken to mean that all people with the putative disease genotype also have manifest lipodystrophy as defined in Row 1, UNLESS they are in likely negative energy balance (undernourished or engaging in high levels of activity) or are prepubertal, when lipodystrophy may not be manifest in thin children.

^f^ Supportive biological evidence often various among different models (e.g., among different cellular systems, different animal strains, or between cells and animals). In this case evidence from any system is judged supportive unless discordant findings in the same system have been reported).

A central feature of the framework is the prioritization of human evidence over biological evidence derived from model systems. This reflects extensive experience in lipodystrophy, where some genes produce striking adipose phenotypes in animal models without causing lipodystrophy in humans [29, 30], whereas several well‐established human lipodystrophy syndromes are incompletely reproduced experimentally [31, 32, 33]. Biological evidence, therefore, provides important mechanistic support but does not determine classification in the absence of convincing human evidence.

The resulting classification system generates a dynamic hierarchy ranging from confirmed (**human evidence H1**) to refuted (**H5**) gene–disease associations (Table 1). Importantly, categories are not intended to be static. As additional clinical, genetic, and biological evidence emerges, disorders may move between categories, allowing the framework to evolve alongside advances in lipodystrophy research.

## Application of the framework to representative disorders

Application of the framework to representative gene–disease associations illustrates how integration of human phenotypic, genetic, and biological evidence yields different levels of confidence in proposed lipodystrophy syndromes (Fig. 2 and Table 2). Detailed gene‐specific scoring according to the clinical genome (ClinGen) framework is shown as a comparator in Table S1.

**Fig. 2:**
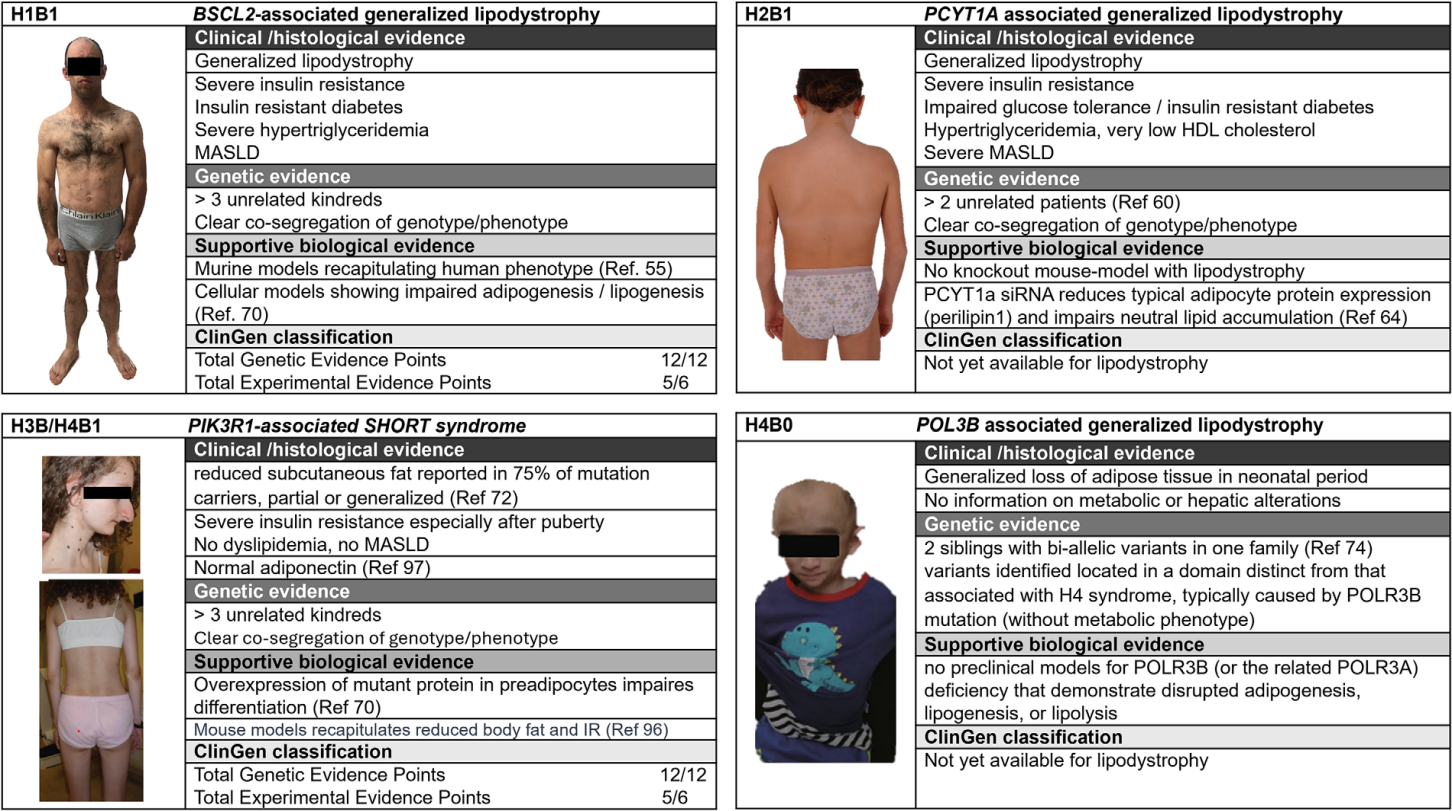
Four examples of the different categories (H1–H4). The male patient living with BSCL2‐associated generalized lipodystrophy was diagnosed at 15 years of age; the photo was taken at 36 years of age. The male patient living with PCYT1A‐associated generalized lipodystrophy was diagnosed at 10 years of age; the photo was taken at 10 years of age. The female patient living with PIK3R1‐associated SHORT syndrome was diagnosed at 12 years of age; the photo was taken at 13 years of age. The male patient living with POL3B‐associated generalized lipodystrophy was clinically diagnosed to have a progeria shortly after birth. The photo was taken at 6 years of age. All patients or their caretakers gave informed consent for the publication of the photos shown here. Photos for H3B/H4B1 have been adapted to the purpose of this publication. They were published previously (Ref. [72]; Creative Commons Attribution 4.0 International License; http://creativecommons.org/licenses/by/4.0/). The photo for H4B0 has been adapted to the purpose of this publication. It was published previously (Ref. [74]; Creative Commons Attribution 4.0 International License; http://creativecommons.org/licenses/by/4.0/). B, supportive biological evidence; BSCL2, lipid droplet biogenesis associated, seipin; H, human evidence (clinical and genetic evidence); HDL, high density lipoprotein; MASLD, metabolic dysfunction‐associated steatotic liver disease; PCYT1A, choline‐phosphate cytidylyltransferase 1A; PIK3R1, phosphoinositide‐3‐kinase regulatory subunit 1; POLR3B, RNA polymerase III subunit B.

**Table 2:** Representative examples of monogenic disorders classified using the ECLip framework.

Category	Representative disorder	Key human evidence	Supporting biological evidence	Illustrative message
H1B1	*BSCL2*‐associated congenital generalized lipodystrophy	Multiple unrelated kindreds; classical generalized lipodystrophy; severe insulin resistance, dyslipidemia and MASLD	Robust cellular and murine models support impaired adipose development and function	Definitive monogenic lipodystrophy
H1B1	*CAV1*‐associated generalized lipodystrophy	Initially limited but subsequently strengthened by additional affected individuals and convincing clinical phenotype	Murine and cellular evidence support adipocyte dysfunction	Example of upward reclassification as evidence accumulates
H2B1	*PCYT1A*‐associated lipodystrophy	Convincing generalized lipodystrophy phenotype in a limited number of affected individuals	Experimental evidence supports impaired lipid droplet/adipocyte biology	Probable lipodystrophy requiring further replication
H3AB1	*CIDEC*‐associated lipodystrophy	Very limited number of affected individuals with convincing partial lipodystrophy and adipose pathology	Strong biological plausibility and adipocyte lipid droplet phenotype	Biologically compelling but numerically fragile association
H3BB1	*PIK3R1*‐associated SHORT syndrome	Reduced adipose tissue reported, but without unequivocal adipose failure; no consistent dyslipidemia or MASLD	Strong genetic and experimental evidence	Distinguishes reduced adiposity from true lipodystrophy
H4B0	*POLR3B*‐associated Wiedemann–Rautenstrauch syndrome	Generalized fat loss reported, but insufficient metabolic or hepatic characterization	No direct biological evidence supporting adipose pathology	Insufficient current evidence for lipodystrophy
H5B1	*LPIN1*‐associated rhabdomyolysis	Human biallelic *LPIN1* variants cause recurrent rhabdomyolysis, with no convincing human lipodystrophy phenotype	Murine models show severe lipodystrophy‐like phenotype	Refuted human lipodystrophy despite strong model‐system evidence

Abbreviations: B, supportive biological evidence; *BSCL2*, lipid droplet biogenesis associated, seipin; *CAV1*, caveolin 1; *CIDEC*, cell death inducing DFFA like effector c; H, human evidence (clinical and genetic evidence); *PCYT1A*, choline‐phosphate cytidylyltransferase 1A; *PIK3R1*, phosphoinositide‐3‐kinase regulatory subunit 1; *POLR3B*, RNA polymerase III subunit B; SHORT syndrome, Short stature, Hyperextensibility, Ocular depression, Rieger anomaly, Teething delay; *LPIN1*, lipin 1.

At the highest level of confidence (H1), disorders, such as *BSCL2*‐associated congenital generalized lipodystrophy [16], fulfill all major criteria for causality. Multiple unrelated affected kindreds have been reported, genotype–phenotype co‐segregation is robust, the clinical phenotype is characteristic, and extensive biological evidence supports a direct role in adipose tissue development and function [52, 53, 54, 55, 56]. Such disorders are widely accepted as definitive monogenic lipodystrophies.

The framework also captures how evidence may strengthen over time. *CAV1*‐associated lipodystrophy illustrates this particularly well: The index human report described a single individual [57], but subsequent identification of additional affected people [58], together with convergent biological evidence [59], progressively consolidated the association and moved the disorder into a higher confidence category.

Many other candidate associations may remain, similarly, in intermediate categories until further replication or more detailed phenotyping becomes available. Disorders, such as *PCYT1A*‐associated lipodystrophy [60] and *CIDEC*‐associated lipodystrophy [33], illustrate this intermediate space. In both, the clinical phenotype and biological rationale are mechanistically highly plausible [61, 62, 63, 64, 65, 66], but the number of reported affected individuals remains limited [67]. The framework therefore recognizes these entities as probable or possible lipodystrophies rather than definitive disorders, while allowing reclassification as additional evidence accumulates.

*PIK3R1*‐associated SHORT syndrome [68, 69] highlights a different challenge. Although genetic and biological evidence are strong [70, 71], the available human data do not yet demonstrate unequivocal adipose tissue failure according to the proposed definition [71, 72, 73]. This distinction is important because reduced adipose tissue mass alone is insufficient to establish lipodystrophy.

*POLR3B*‐associated Wiedemann–Rautenstrauch syndrome illustrates yet another evidential scenario. Generalized adipose tissue deficiency has been reported in a small number of affected individuals, raising the possibility of lipodystrophy [74]. However, available clinical descriptions provide limited information regarding metabolic manifestations or adipose tissue dysfunction, and there is currently no direct biological evidence linking *POLR3B* deficiency to impaired adipose tissue development or function. Consequently, the disorder is currently classified as H4B0, reflecting insufficient evidence to establish a diagnosis of lipodystrophy according to the proposed framework. Importantly, this classification should not be interpreted as evidence against a role for adipose tissue pathology, but rather as highlighting the need for more comprehensive phenotypic and mechanistic data.

*LPIN1* provides the converse lesson. Murine *Lpin1* deficiency produces a severe lipodystrophy‐like phenotype, and strong biological evidence links lipin‐1 to adipose biology [29, 75, 76]. However, biallelic *LPIN1* variants in humans, identified after seminal experimental studies in mice, cause recurrent rhabdomyolysis rather than lipodystrophy [77], and available human data do not support primary adipose tissue failure [30]. *LPIN1* is therefore classified as H5/B1, illustrating why biological evidence is considered supportive rather than determinative in the proposed hierarchy (for name of genes see list of abbreviations).

These examples illustrate a central feature of the ECLip framework: Disease classification is driven primarily by evidence derived from affected humans, with biological evidence providing mechanistic support. The framework accommodates variable penetrance, expressivity, and imperfect model‐system fidelity and provides a transparent mechanism through which gene–disease associations can be re‐evaluated and reclassified as knowledge advances.

## Clinical and research implications

Setting out a formal definition and a framework for evidence evaluation for monogenic lipodystrophy seeks to address two major challenges confronting those engaged in clinical care and/or translational research. These are the poor awareness of many clinicians of lipodystrophy, leading to diagnostic delays commonly of years or even decades, and second the imprecise use of the “lipodystrophy” label even amongst scientists and clinicians who are aware of the disease entity.

Addressing the long diagnostic “odyssey” for those with lipodystrophy will be of most immediate clinical impact. Lacking a precise diagnosis and connection to appropriate peer support and advocacy itself engenders significant psychological morbidity, as in any rare disease. However, the specific harm caused by delayed diagnosis of lipodystrophy extends far beyond this. Targeted management of lipodystrophy can substantially reduce morbidity from major end‐organ complications, including advanced liver disease, microvascular complications of diabetes, atherosclerosis, acute pancreatitis, and complicated pregnancy.

Treatment of lipodystrophy is similar in principle to the treatment of obesity with complications, including dietary optimization, exercise counseling, intensive management of diabetes and dyslipidemia, and surveillance for hepatic, cardiovascular, and reproductive complications. Crucially, body mass index is of greatly reduced importance in deciding choice of therapy. Before a diagnosis of lipodystrophy is made, many patients may be denied appropriate therapy due to failure to appreciate the importance of offloading adipose tissue metabolically even in the absence of obesity. Recognizing lipodystrophy also opens the possibility of more advanced therapy with metreleptin—an effective disease‐specific therapy licensed for patients with generalized lipodystrophy and also for selected individuals with partial lipodystrophy in Europe [2]. Emerging therapies directed at metabolic dysfunction may further improve outcomes. Accurate classification is therefore not merely an academic exercise but has direct implications for prognosis, therapeutic access, and long‐term disease management.

The current work addresses diagnostic delay by offering a precise definition of lipodystrophy that can be disseminated widely amongst the diverse clinical specialists to whom people with lipodystrophy first present, increasing awareness and consideration of lipodystrophy. The guidance in the definition on the difference between leanness without lipodystrophy and lipodystrophy will, moreover, aid specificity of diagnosis.

The second contribution of the new proposals is to suggest a classification framework for proposed monogenic lipodystrophy subtypes that stratifies them according to the strength of the underpinning evidence. This will have a longer term, but no less important, impact for the field. The suggested framework offers a dynamic scheme within which new proposed disease entities can advance as evidence accumulates with time. It places primacy on human evidence but does not exclude either emerging evidence from model systems that may predict human forms of lipodystrophy yet to be described or clear examples of mismatch between animal models and humans that may be scientifically informative.

Our proposed definition is applicable to all lipodystrophy subtypes, but our classification schema currently addresses only monogenic lipodystrophy. It will be readily customizable to address acquired lipodystrophies, with genetic evidence supplanted by grading of evidence for the acquired pathogenic process—the subject of future work. We have also excluded polygenic/oligogenic FPLD1 from the schema. FPLD1 is a crucial “bridge” to common forms of unhealthy obesity but effectively represents the end of the population spectrum of adipose topography, and its demarcation is arbitrary and still the subject of debate. We note, however, that any form of lipodystrophy initially classified as FPLD1 may come within the scope of the current framework if and when a putatively pathogenic gene variant is described in affected individuals.

The current focus on novel candidate forms of monogenic lipodystrophy is timely, as claims of monogenic etiologies for lipodystrophy have proliferated in the era of widespread diagnostic next‐generation sequencing. This is not a concern limited to monogenic lipodystrophy and has been widely discussed across the clinical genetics community [78]. A particular concern has been “pollution” of the literature with poorly substantiated gene–disease associations, potentially leading to inappropriate reporting of genetic results to patients. One major response has been creation of an open‐access resource by the National Institutes of Health (NIH)‐funded Clinical Genome Resource (ClinGen), aimed at improving definition of clinically relevant genes based on standardized evidence assessment [79]. The classification scheme used to do this is based on a semiquantitative assessment of the strength of evidence for a gene–disease relationship [79], and its reproducibility has been validated across different curators for a subset of genes [79].

The classification framework does not seek to compete with the ClinGen process. Rather, it offers an approach that can ultimately be extended to incorporate acquired and polygenic disease subtypes as well as monogenic entities. The evaluation of genetic and experimental evidence is consciously less detailed than the ClinGen approach, but more nimble, meaning it can more plausibly stay abreast of the rapid pace of genetic research and discovery in lipodystrophy. Our approach also pays greater attention to the complexities of penetrance and expressivity, which are particularly challenging for lipodystrophy compared to other developmental disorders.

The approach adopted is also distinct from ClinGen in its hierarchical approach to evaluation, with human clinical evidence at the apex, followed by genetic and then experimental evidence. These tiers of evidence are kept separate in the ultimate classification, with the consequence that interesting and potentially informative mismatches between different tiers of evidence are explicit. In contrast, the ClinGen approach, although computing genetic and experimental scores, ultimately yields a single score that encompasses both. In a condition such as lipodystrophy, where there is exquisite interaction between environmental stressors and genotype, and where lipodystrophy is often only part of a wider developmental syndrome, this simple approach leads to loss of nuance that may be clinically relevant.

## Conclusion

In summary, we present a consensus definition of lipodystrophy developed by an international expert group spanning 14 countries. The definition emphasizes that anatomical adipose deficiency alone is insufficient for diagnosis; evidence of adipose functional insufficiency, most commonly manifested as a metabolic profile discordant with the degree of leanness, is also required to diagnose lipodystrophy. Building on this definition, we propose a hierarchical classification framework for monogenic lipodystrophy that stratifies candidate subtypes by the strength of human phenotypic, genetic, and experimental evidence. By placing human clinical evidence at the apex, the framework resists misclassification driven by compelling but imperfectly predictive animal models, while remaining dynamic enough to accommodate new evidence as it emerges. Together, these tools are intended to sharpen clinical recognition of lipodystrophy across the many specialties to which affected people present and to bring greater rigor to the rapidly expanding genetic literature on this condition.

## Author contributions

**Robert K. Semple**: Writing—original draft; conceptualization; investigation; methodology; validation; data curation; project administration; visualization. **Martin Wabitsch**: Data curation; project administration; writing—review and editing; investigation. **Baris Akinci**: Writing—review and editing; data curation; investigation. **Saif Alyaarubi**: Data curation; writing—review and editing; investigation. **Marjoleine F. Broekema**: Writing—review and editing; data curation; investigation. **Rebecca J. Brown**: Data curation; writing—review and editing; investigation. **Giovanni Ceccarini**: Writing—review and editing; data curation; investigation. **Carolina Cecchetti**: Writing—review and editing; data curation; investigation. **Solaf M. Elsayed**: Data curation; writing—review and editing; investigation. **Antía Fernández‐Pombo**: Writing—review and editing; data curation; investigation. **Alessandra Gambineri**: Data curation; writing—review and editing; investigation. **Ingrid M. Jazet**: Writing—review and editing; data curation; investigation. **Lotte Kleinendorst**: Data curation; writing—review and editing; investigation. **Konstanze Miehle**: Writing—review and editing; data curation; investigation. **Elif A. Oral**: Data curation; writing—review and editing; investigation. **Flavia Prodam**: Writing—review and editing; data curation; investigation. **Ferruccio Santini**: Data curation; writing—review and editing; investigation. **David B. Savage**: Investigation; writing—review and editing; data curation. **Nadia Shaukat Ali**: Writing—review and editing; data curation; investigation. **Ekaterina Sorkina**: Data curation; writing—review and editing; investigation. **Magdalena Szopa**: Writing—review and editing; data curation; investigation. **Corinne Vigouroux**: Data curation; writing—review and editing; investigation. **Marie‐Christine Vantyghem**: Data curation; writing—review and editing; investigation. **Camille Vatier**: Writing—review and editing; data curation; investigation. **Julia von Schnurbein**: Visualization; validation; methodology; project administration; investigation. **David Araújo‐Vilar**: Conceptualization; visualization; validation; methodology; project administration; data curation; investigation; supervision; formal analysis.

## Conflict of interest statement

B.A. has served as a consultant, board member, steering committee member, and/or speaker to Amryt (now Chiesi), Regeneron, Astra Zeneca, Novo Nordisk, Boehringer Ingelheim, Sanofi, Bilim Ilac, ARIS, Servier, and Eczacibasi. R.J.B. serves as principal investigator for Regeneron‐sponsored studies of mibavademab in lipodystrophy, for which her institution has received funding. She has received a grant from Pfizer providing free drug (pegvisomant) for a study. She conducts investigator‐initiated studies for which Chiesi provides free drug (metreleptin). She has also received research funding from Marea for physiology studies. J.v.S. and M.W. have received fees from Amryt (now Chiesi) and Rhythm or scientific advice, travels, and speakers’ fees and are investigators in sponsor‐initiated trials for Regeneron and Chiesi. D.A.‐V. has received fees from Amryt (Chiesi) and Regeneron for scientific advice, travels, conference registrations, and research grants. R.K.S. has received fees from Amryt (now Chiesi) for scientific consultancy and speaker fees for sponsored talks at conferences. F.S. received a consultancy fee from Amryt (Chiesi); during the last 2 years he has run projects for and/or served as a consultant, board member, steering committee member, and/or speaker for Amryt (Chiesi), Novo Nordisk, Eli Lilly, Pfizer, Amgen, and Roche. Co.V. received personal fees for lectures from Amryt (Chiesi), Sanofi, and Gilead and personal support for attending meetings from Chiesi and Asten‐Santé and is investigator in sponsor‐initiated trials for Regeneron and Chiesi. G.C. has received consulting fees from Chiesi and Rhythm, lecturing fees and travel grants from Rhythm, Chiesi, Novo Nordisk, and Lilly. M.C.V. is investigator in sponsor‐initiated trials for Regeneron and Chiesi and received travel grants Ascendis. N.S.A. received travel grants from Rhythm. Ca.V. received personal fees from lecturers from Sanofi, Amryt (Chiesi), Astra Zenecca, Mennarini, Lilly, and Novo Nordisk, travel grants from Sanofi and Lilly, was participating on data safety/advisory boards for Amryt (Chiesi), Sanofi, and Menarini and is investigator in sponsor‐initiated trials for Regeneron and Chiesi. C.C. received travel grants from Chiesi. F.P. received lecture payments from Chiesi, Novo Nordisk, Menarini, and Novartis, travel grants from Chiesi and Novo Nordisk and was on advisory boards for those two companies as well. K.M. received consulting and lecture fees and travel grants from Chiesi. M.S. received lecture fees and travel grants from Eli Lilly and Boehringer Ingelheim. S.M.E. received personal lecture fees from New bridge pharma. E.A.O. has intellectual property and receives royalty fees from metreleptin use in lipodystrophy, grant support, consulting fees, and clinical trial support from Chiesi, Regeneron, and Ionis Pharmaceuticals, clinical trial support from Rhythm, Novo Nordisk, Morphic Medical, and Fractyl, and grant support from Rejuvenate Bio. D.B.S. participated on data safety monitoring or advisory boards for Regeneron.

## Supporting information




**Table S1** Examples of Lipodystrophy subtypes according to the new ECLip categories.

## Data Availability

The data that support the findings of this study are available on request from the corresponding author. The data are not publicly available due to privacy or ethical restrictions.
