# Supplementary material for: European Consortium for Lipodystrophies consensus definition and classification framework for monogenic lipodystrophy
Source: J Intern Med. 2026 Sep 1;300(6):725–42. doi: 10.1111/joim.70157 (PMC13741812; doi:10.1111/joim.70157)
Supplement: Supplementary file 1 [file JOIM-300-725-s001.docx]

**Supplemental Table 1. Examples of Lipodystrophy subtypes according to the new ECLip categories**

| human evidence (clinical and genetic) (H) /  supportive biological evidence (B) | ClinGen classification | |
| --- | --- | --- |
| **H1: confirmed** | **Total Genetic Evidence Points (Maximum 12)** | **Total Experimental Evidence Points (Maximum 6)** |
| **H1B1** |  |  |
| *BSCL2*-associated generalized lipodystrophy (AR) | 12 | 5 |
| *CAV1*-associated generalized lipodystrophy (AR) | 7 | 6 |
| **H1B2** |  |  |
| *POLD1*-associated MDPL syndrome (AD) | 12 | 1 |
| **H1B0** |  |  |
| *CAV1*-associated progeroid lipodystrophy (AD) | 1.6 | 0 |
| **H2: probable** |  |  |
| **H2B1** |  |  |
| *PCYT1A*-associated lipodystrophy (AR) | NA | NA |
| **H2B2** |  |  |
| *BANF1*-associated Néstor-Guillermo progeria syndrome (AR) | 3 | 1 |
| **H3: possible** |  |  |
| **H3AB1** |  |  |
| *CIDEC*-associated lipodystrophy (AR) | NA | NA |
| **H3AB2** |  |  |
| *AIRE*-associated Lipodystrophy and PGA syndrome type 1 (AR) | 12 | 5.5 |
| **H3BB1** |  |  |
| *PIK3R1*-associated SHORT syndrome (AD) | 12 | 6 |
| **H3BB2** |  |  |
| *PSMB10*-associated autoinflammatory syndrome (AR) | 1.5 | 3 |
| **H4 - No Evidence** |  |  |
| *POLR3A*-associated Wiedemann-Rautenstrauch syndrome (AR) | 11.4 | 2 |
| *POLR3B*-associated Wiedemann-Rautenstrauch syndrome (AR) | NA | NA |
| **H5 - Refuted** |  |  |
| *LPIN* | 7.5 | 4.5 |

The table displays typical examples of monogenic lipodystrophy syndrome categorized according to the new ECLip categorization.

Human evidence refers to published cases of patients with evidence of lipodystrophy taking into account anatomical evidence of lack of adipose tissue, metabolic evidence of adipose tissue failure or histological changes in adipocytes and lastly also genetic evidence (number of published patients and co-segregation in families).

Supportive biological evidence refers to animal model(s) recapitulating human phenotype and/or cellular model(s) showing impaired adipogenesis/ lipogenesis /lipolysis.

For comparison, the available ClinGen classification is shown alongside where available

B = supportive biological evidence; H = human evidence (clinical and genetic evidence); CinGen = Clinical Genome; BSCL2 = lipid droplet biogenesis associated, seipin; AR = Autosomal recessive; CAV1 =caveolin 1; POLD1 = DNA polymerase delta 1, catalytic subunit; AD = Autosomal dominant; PCYT1A = choline-phosphate cytidylyltransferase 1A, N/A = not available; BANF1 = barrier to autointegration nuclear assembly factor 1; CIDEC = cell death inducing DFFA like effector c; AIRE = autoimmune regulator; SHORT syndrome = Short stature, Hyperextensibility, Ocular depression, Rieger anomaly, Teething delay; PIK3R1 = phosphoinositide-3-kinase regulatory subunit 1; PSMB10 = proteasome 20S subunit beta 10; POLR3 A or B = RNA polymerase III subunit A or B; LPIN1 = lipin 1;
